# Genetic manipulation of longevity-related genes as a tool to regulate yeast life span and metabolite production during winemaking

**DOI:** 10.1186/1475-2859-12-1

**Published:** 2013-01-02

**Authors:** Helena Orozco, Emilia Matallana, Agustín Aranda

**Affiliations:** 1 Departamento de Biotecnología, Instituto de Agroquímica y Tecnología de Alimentos-CSIC, Av. Agustín Escardino, 7, Paterna 46980, Spain; 2 Departament de Bioquímica i Biologia Molecular, Universitat de València, Avda. Dr Moliner 50, Burjassot, 46100, Spain

**Keywords:** Yeast, Chronological aging, Stress, Ethanol, Sirtuins, *HST3*, *PUB1*

## Abstract

**Background:**

Yeast viability and vitality are essential for different industrial processes where the yeast *Saccharomyces cerevisiae* is used as a biotechnological tool. Therefore, the decline of yeast biological functions during aging may compromise their successful biotechnological use. Life span is controlled by a variety of molecular mechanisms, many of which are connected to stress tolerance and genomic stability, although the metabolic status of a cell has proven a main factor affecting its longevity. Acetic acid and ethanol accumulation shorten chronological life span (CLS), while glycerol extends it.

**Results:**

Different age-related gene classes have been modified by deletion or overexpression to test their role in longevity and metabolism. Overexpression of histone deacetylase *SIR2* extends CLS and reduces acetate production, while overexpression of *SIR2* homolog *HST3* shortens CLS, increases the ethanol level, and reduces acetic acid production. *HST3* overexpression also enhances ethanol tolerance. Increasing tolerance to oxidative stress by superoxide dismutase *SOD2* overexpression has only a moderate positive effect on CLS. CLS during grape juice fermentation has also been studied for mutants on several mRNA binding proteins that are regulators of gene expression at the posttranscriptional level; we found that *NGR1* and *UTH4* deletions decrease CLS, while *PUF3* and *PUB1* deletions increase it. Besides, the *pub1*Δ mutation increases glycerol production and blocks stress granule formation during grape juice fermentation. Surprisingly, factors relating to apoptosis, such as caspase Yca1 or apoptosis-inducing factor Aif1, play a positive role in yeast longevity during winemaking as their deletions shorten CLS.

**Conclusions:**

Manipulation of regulators of gene expression at both transcriptional (i.e., sirtuins) and posttranscriptional (i.e., mRNA binding protein Pub1) levels allows to modulate yeast life span during its biotechnological use. Due to links between aging and metabolism, it also influences the production profile of metabolites of industrial relevance.

## Background

Biological aging, or senescence, is defined as the degradation of the biological function that accompanies the passage of time [1], and it is a process that affects cells, individuals and populations. Therefore, the performance of microorganisms during their biotechnological use can be affected by the life span of the members of a population for a given condition. For instance, baker’s yeast *Saccharomyces cerevisiae* has two aging models [2]. Replicative life span (RLS) is the number of daughter cells produced by a mother cell before senescence, which can be easily visualized due to the asymmetric nature of *S*. *cerevisiae* cell divisions. This fixed amount of cell divisions becomes relevant when there is continuous growth, for instance during biomass propagation, beer production [3] or sugar cane fermentation to obtain biofuel [4], where the yeast biomass produced at the end of the processes is re-used to inoculate new fermentations. Chronological life span (CLS) is defined by how long a yeast cell can survive in a non dividing, quiescence-like state. This aging model is more relevant when fermentation is carried out mostly by non dividing cells, which is the case of grape juice fermentation in winemaking [5]. Modern winemaking practices include inoculation of grape juice with starter cultures in the form of active dry yeasts. Under these conditions, the yeast growth phase implies only 4-6 cycles of cell division, far from the 20 divisions of the mean maximal RLS of natural isolates [6]. Therefore RLS is not a limiting factor for yeast performance, unlike viability in the stationary phase which is 3-4 times longer than the growth phase under winemaking conditions [5]. “Sur lies” aging refers to aging wine on yeast lees (death cells). During this period, cells undergo autolysis by releasing enzymes that change the wine composition to generate desirable organoleptic properties [7]. Release of intracellular components after cell death and lysis may also influence the growth of microorganisms, and they may be positive for winemaking, such as lactic acid bacteria involved in malolactic fermentation [8], or negative; e.g., growth of spoiling microorganisms, such as other yeasts or acetic bacteria. The environmental factors involved in CLS during winemaking have been studied in our laboratory, and it is clear that the high concentration of two-carbon metabolites produced by yeast metabolism, such as ethanol, acetic acid and acetaldehyde, are key factors for longevity [9].

The traditional biochemical way of describing senescence has been the free radical theory of aging, established in 1956 [10]. Relevance of the oxygen reactive species generated by metabolism or by exogenous oxidants on life span has been described in many organisms, including yeast [11]. In a previous work, we demonstrated that tolerance to oxidative stress correlates to CLS in wine yeasts [12]. However, there is an increasing challenge for this conventional conception of aging, and many authors interpret oxidative damage as a consequence, and not a cause, of aging [13].

In any case, it is clear that aging is a complex process involving a variety of molecular mechanisms, many of which have been discovered in yeast [2]. The first screening for yeast mutants with increased RLS identified four genes known as *UTH14*[14]. One of them, *UTH4*, codes for an RNA binding protein related to the re-localization of another aging protein, Uth2, from the telomere to the nucleolus [15]. Uth2 happens to be Sir4, a member of a complex ligated to genomic stability in which sirtuin Sir2 plays an important role. Sirtuins are NAD^+^-dependent histone deacetylases [16]. There are five yeast sirtuins, Sir2 (which gives its name to the family) and Hst1-4. Sir2 has been defined as an RLS extending factor given its role in heterochromatin formation, therefore promoting genome stability. The silencing of sub-telomeric regions by the action of Sir2 seems to be a key factor in replicative life span [17]. However, the role of Sir2 in CLS seems to be the opposite as Sir2 blocks CLS by repressing the activity of alcohol dehydrogenase 2, therefore increasing the ethanol concentration in the stationary phase, which acts a pro-aging factor [18]. It has been reported that direct acetylation/deacetylation is a posttranslational form of metabolic regulation. In yeast, the regulation of the gluconeogenic Pck1 enzyme by Sir2 has been fully described [19]. The fact that this family of deacetylases consumes a NAD^+^ molecule in their enzymatic reaction makes them act as metabolic sensors, and global analyses suggest that Sir2 controls a complex metabolic network [20]. In addition to ethanol, two other metabolites produced during fermentation have been linked to CLS under laboratory conditions. Acidification by acetic acid production is a very important cause of aging in laboratory conditions [21,22], while glycerol accumulation extends longevity [23].

A consensus has been reached that low food intake without malnutrition, i.e. dietary restriction (DR), is the only intervention that extends life span from yeast to mammals [24]. Sir2 has been described as a key factor in extending RLS by glucose restriction [25]. However, this hypothesis has been recently challenged and it is now the center of bitter controversy [26]. Moreover, the role of Sir2 overexpression in aging in flies and worms has proved not to be as strong as previously assumed [27]. Previously, we proved that sirtuin deletion in wine yeasts affects CLS, often in a growth medium-dependent way [28]. For instance, Sir2 deletion extends CLS under laboratory conditions, but shortens it during grape juice fermentation. Besides, sirtuin deletion affects metabolite production during winemaking as *sir2*Δ mutants produce more ethanol and less acetic acid, while *hst3*Δ has the opposite effect. Therefore, sirtuin manipulation can be envisaged as an efficient way to control metabolism.

Yeast death during chronological aging under laboratory conditions has been related to similar features to programmed cell death or apoptosis in higher organisms [29]. The benefits of apoptosis on single cell microorganisms have to be considered in the population survival context. The “altruistic aging” concept suggests that some cells may die and then nutrients to the rest of the colony are released. In this kind of aging, the oxidative stress caused by the superoxide anion is important, while the action of superoxide dismutases, like Sod2, blocks its action [30]. Many molecular components in apoptotic machinery are present in *S*. *cerevisiae*, such as caspase Yca1 and mitochondrial apoptosis-inducing factor Aif1 [31].

Our aim in this work was to manipulate a variety of genetic determinants of cell aging in order to study their influence on cell viability during grape juice fermentation and their impact on metabolism under this condition. The overexpressions of sirtuins *SIR2* and *HST3* prove to be an efficient tool to manipulate longevity and metabolite production. Increasing doses of *HST3* produce more ethanol and less acetic acid, while the overexpression of *SIR2* extends longevity. Manipulation of the oxidative stress machinery represented by the gene coding for superoxide dismutase 2 has only a moderate impact on life span, while deletion of apoptosis factors unexpectedly shortened CLS. We studied the role of several mRNA binding proteins as potential posttranscriptional regulators, and identified *PUB1* as the gene whose deletion increases both CLS and glycerol production under winemaking conditions. Therefore, life span is closely linked to metabolism during grape juice fermentation by wine yeasts.

## Results and discussion

### Modulation of life span by the overexpression of sirtuin genes

**Figure 1:**
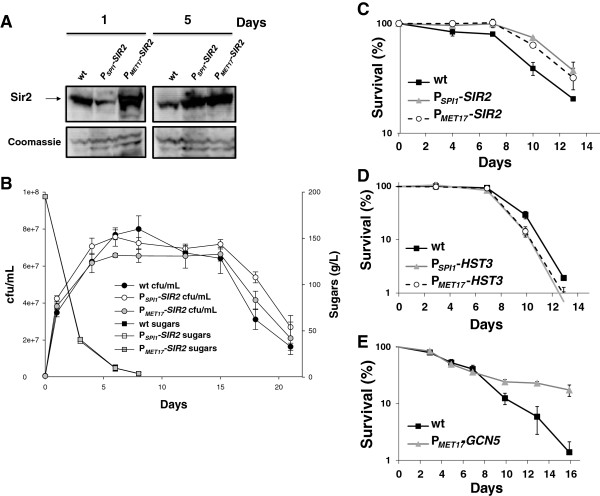
**Sirtuin gene overexpression modulates life span under winemaking conditions. A**) Western blot showing Sir2 levels on days 1 and 5 of natural grape juice fermentation. Wild-type commercial strain L2056 and its derivatives with *SIR2 *under the control of promoters *SPI1 *and *MET17 *are included. **B**) Natural grape juice fermentations of the *SIR2*-overexpressing strains described in Panel **A**. Cell viability over time was measured as cfu/mL and fermentation evolution was followed by measuring sugar consumption. **C**) Survival curve of the *SIR2*-overexpressing strains under winemaking conditions. Cell viability on day 8 of Panel **B** was taken as 100% survival. The survival curves of the *HST3*- (**D**) and *GCN5*- (**E**) overexpressing strains. Other conditions as in Panel **C**. Experiments were done in triplicate and the mean and standard deviation are provided.

In order to test the impact of sirtuin overexpression under winemaking conditions, the *SIR2* gene was expressed under the control of two heterologous promoters following the promoter-replacement strategy developed in our laboratory [32]. Two promoters with different expression profiles during grape juice fermentation [33] were chosen. *SPI1* is a stationary phase specific gene that has been described to be induced at late fermentation stages [33,34]. *MET17* is a gene involved in methionine biosynthesis which is activated during the first days of fermentation [33]. Overexpression was performed in wine yeast strain L2056, a diploid for the *SIR2* gene [28], by promoter replacement on one of its two copies. Transformants were used to perform natural grape juice fermentation, and yeast proteins were extracted 1 or 5 days after inoculation for Sir2 detection by using a specific antibody (Figure 1A). On day 1, the *SPI1* promoter was repressed if compared to the parental strain, but the *MET17* promoter caused a higher *SIR2* expression, as expected. On day 5, when cells have entered stationary phase (see Figure 1B) and less than one fourth of initial sugars were present, *SPI1* led to a higher Sir2 expression than in the wild-type strain, as expected. Unexpectedly however, *MET17* also accomplished this, indicating that, under our conditions, either this gene showed a high expression throughout fermentation or high levels of the Sir2 protein accumulated at the beginning of fermentation to remain stable for a long time. In any case, the overexpression strategy via promoter replacement rendered the expected result of increasing Sir2 levels. Hence, it is a successful way of increasing the dose of selected proteins during grape juice fermentation on wine yeast.

Next we analyzed the evolution of natural grape juice fermentation carried out by the constructed strains (Figure 1B). Growth and death were monitored by counting the viable cells able to form a colony when plated in rich medium and expressed as cfu (colony forming units) per mL. Fermentation progress was followed by measuring the amount of remaining sugars. All the manipulated strains were able to finish fermentation and to consume all the sugars in a similar time. Therefore, their fermentative capacity was not challenged. *SIR2* overexpression under the *SPI1* promoter did not interfere with growth (which was slightly faster than for the wild-type strain). Cells reached a similar final density, but viability remained higher than the parental strain after sugar depletion. If maximal cell viability (in this case, day 8 for the wild-type strain) was taken as 100% survival, a plot showing the death profile was obtained (Figure 1C). In this case, it can be clearly seen that the overexpression of *SIR2* extended CLS under winemaking conditions, which is the exact opposite effect to that produced by *SIR2* deletion [28]. The *SIR2* expression under the *MET17* promoter slightly reduced final cell density (Figure 1B), but also extended CLS (Figure 1C). This result indicates that Sir2 plays a positive role on CLS under winemaking conditions, which is the equivalent to the positive role on RLS under laboratory conditions [35]. Therefore, despite the controversial role in aging of the Sir2 overexpression in animal models [27], it plays a role in longevity in *S*. *cerevisiae* as similar results have been obtained in different genetic backgrounds and experimental conditions.

We performed the same overexpression strategy on another member of the sirtuin family, *HST3*, whose deletion had the opposite effect to *SIR2* deletion in CLS and ethanol production [28]. Its deletion led to an increase in CLS and a slightly smaller amount of ethanol. The survival curve (similar to that described in Figure 1C) under fermentation conditions showed that the over-expression of *HST3* with both promoters leads to a slightly shortened CLS (Figure 1D), the opposite effect to that observed for the *SIR2* overexpression, and is also the reverse effect to that induced by *HST3* deletion. Fermentation capacity did not alter (data not shown); therefore, wine yeast chronological longevity under winemaking conditions can be modulated by the deletion or overexpression of different sirtuin family members.

Next we overexpressed the gene coding for acetyltransferase Gcn5 under the control of the *MET17* promoter, as it gives high expression through all fermentation (see Figure 1A). *GCN5* deletion caused CLS to extend [28] and, unexpectedly, its up-regulation also extended maximal life span (Figure 1E). As Gcn5 acts with a variety of multiprotein complexes, such as SAGA, SLIK or SALSA [36], its overexpression may alter the amount or proportion of these complexes, thus producing this unexpected impact on longevity as they regulate multiple targets.

### *HST3* overexpression increases ethanol production and tolerance

**Figure 2:**
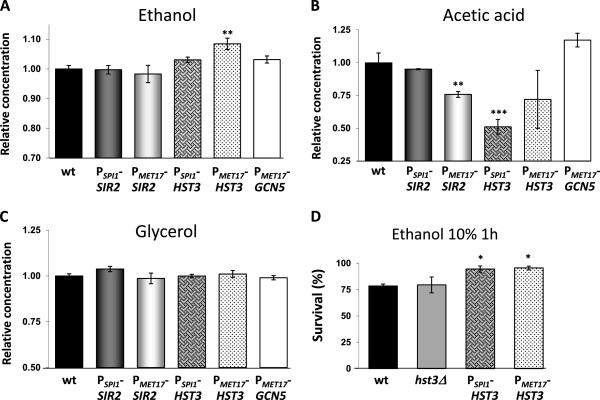
***HST3 *overexpression reduces acetic acid production and enhances ethanol tolerance.** Ethanol (**A**), acetic acid (**B**) and glycerol (**C**) production at the end of fermentation for the overexpressing strains described in Figure 1 in relation to the concentration of each metabolite produced by the parental L2056 strain. **D**) Survival after the incubation of stationary cultures on YPD at 10% ethanol for 1 hour. Cells were diluted, plated on YPD plates and counted before and after stress application. Experiments were done in triplicate and the mean and standard deviation are provided. *p<0.05, **p<0.01, ***p<0.005, unpaired t-test, two-tailed.

Our next objective was to test the effect of these overexpressions on metabolite production at the end of grape juice fermentation (Figure 2). As mentioned earlier, none of these manipulations altered the ability to fully consume sugars under winemaking conditions. We measured three essential metabolites for wine organoleptic properties: ethanol, acetic acid and glycerol. Regarding ethanol production (Figure 2A), only *HST3* overexpression under the *MET17* promoter significantly increased ethanol production. When the *HST3* overexpression was prompted by the *SPI1* promoter, it led to a slight, but not significant, increase in final ethanol production. This result matches the fact that *HST3* deletion reduces ethanol production [28], possibly indicating that ethanol accumulation causes premature aging in these strains (Figure 1C). However, *SIR2* manipulations had no impact on ethanol production, despite the role of the corresponding deletion on ethanol metabolism [28]. To test if the increase of ethanol production in *HST3*-overexpressing strains altered biomass production, we measured the dry biomass after completion of fermentation carried out in synthetic grape juice MS300 (to minimize the effect of solids present in natural grape juice). Both overexpressing strains had a decreased biomass production (Additional file 1: Figure S1A). Ethanol is also increased in this medium, particularly for the *SPI1* promoter-driven *HST3* mutant (Additional file 1: Figure S1B). Therefore *HST3* activity cause a shift of carbon from biomass production to ethanol production.

Acetic acid production was also affected, as expected, by *HST3* overexpression (Figure 2B). *HST3* deletion increased acetic acid production [28], and its overexpression brought about a lower final concentration (although it was significant only in the strain with the *SPI1* promoter-driven *HST3*). A lower acetic acid level did not correlate with the shortened longevity of those strains. Therefore, acetic acid is not the main cause of CLS under winemaking conditions. *SIR2* deletion has been described to cause low acetic acid production, and its overexpression by the *MET17* promoter surprisingly led to a similar phenotype (Figure 2B). It is also striking that *GCN5* overexpression caused no major impact on acetic acid production, (only a slight increase was seen), while its deletion notably increased acetic acid production under several growth conditions [28]. Likely, the multiple targets of those proteins may explain the complex metabolite profile produced by their overexpression, that do not always have the opposite output than their deletion.

As regards glycerol production (Figure 2C), lack of effects contrasts with the increased glycerol production observed in the *gcn5*Δ strain [28]. In fact, none of these genetic alterations was able to significantly change the amount of this relevant metabolite. It is also worth mentioning that none of these genetic manipulations alter acetaldehyde production (data not shown). Therefore the impact of acetylation/deacetylation machinery in metabolite production is complex and difficult to predict, but can highlight interesting strategies to change the metabolic profile of wine, as the case of *HST3* over-expression proves. Although we do not yet have a molecular explanation for the role of Hst3 in metabolic regulation, the combined deletion of *HST3* and its homolog *HST4* in laboratory strains prevents growth on acetate and propionate as a sole carbon source [37], suggesting that yeast sirtuins may act as human or *Salmonella* sirtuins to modulate the action of acetyl-CoA synthetase (Acs). However, there is no evidence of a reversible acetylation involved in Acs regulation, and we detected no change in Acs activity in the *hst3*Δ or *hst4*Δ mutants [28], so the implication of sirtuins in this regulation may be redundant.

Changes in ethanol production may affect yeast cells’ ability to deal with toxicity caused by an accumulation of this metabolite, the main stress condition at the end of wine fermentation. Deletion of sirtuins, including *HST3*, had no impact on ethanol tolerance (Figure 2D and data not shown), but *HST3* overexpression not only increased ethanol production (Figure 2A), but also enhanced tolerance to it (Figure 2D). Despite increased tolerance, *HST3* overexpression shortened CLS (Figure 1D); therefore, an increase in ethanol may be detrimental for CLS, even for these stress-resistance strains. *HST3* transcription was induced by cocoa polyphenols and proved necessary for the protection that these polyphenols offer against oxidative stress [38]. Therefore, sirtuin manipulation could allow to modulate stress tolerance in yeast under natural conditions.

### Superoxide dismutase *SOD2* overexpression has a limited impact on life span during winemaking

**Figure 3:**
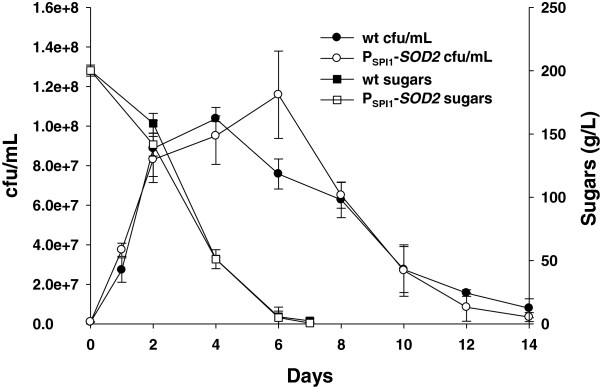
**Superoxide dismutase 2 overexpression has a mild impact on life span. **The *SOD2*-overexpressing strain and its parental strain L2056 were used to inoculate natural grape juice fermentations. Cell viability over time was measured as cfu/mL and fermentation evolution was followed by measuring sugar consumption. Experiments were done in triplicate and the mean and standard deviation are provided.

According to the free radical theory of aging, the induction of anti-oxidant enzymes should contribute to extend life span. One of these enzymes is mitochondrial superoxide dismutase Sod2, which performs an important task in CLS under laboratory conditions [39]. To test the impact of improving oxidative stress tolerance at the stationary phase on winemaking conditions, we expressed the *SOD2* gene under stationary phase promoter *SPI1* in wine yeast L2056. This transformation did not significantly affect grape juice fermentation as sugar consumption profiles were similar for modified and parental strains (Figure 3). Cell growth between strains was also similar, but cell viability increased in the overproducing strain when sugar levels were low. However when sugars were exhausted, both strains showed a similar long-term life span. Therefore, the impact of mitochondrial defense systems on longevity does not seem relevant when sugars are absent, thus mitochondrial activity is assumed to be not glucose-repressed. This indicates that this cellular metabolic situation differs from the stationary phase in standard growth media for laboratory yeast strains. *SOD2* overexpression brought about the opposite effect to the deletion of another mitochondrial antioxidant gene, *RDL2*[40]. In this case, viability before glucose exhaustion dropped and ROS concentration increased. Therefore, these results suggest that mitochondrial activity and anti-oxidant responses are necessary at the end of fermentation when the sugar concentration is low. Yet surprisingly once fermentation has finished, these systems seem to play no role, despite the fact that the absence of sugars is no longer repressing respiration and stress response. It should be taken into account that ethanol is not consumed at the end of vinification, therefore our molecular results indicate that this physiological situation is not the equivalent to that of laboratory strains growing on a non-fermentable carbon source.

### RNA-binding proteins are life span modulators under winemaking conditions

**Figure 4:**
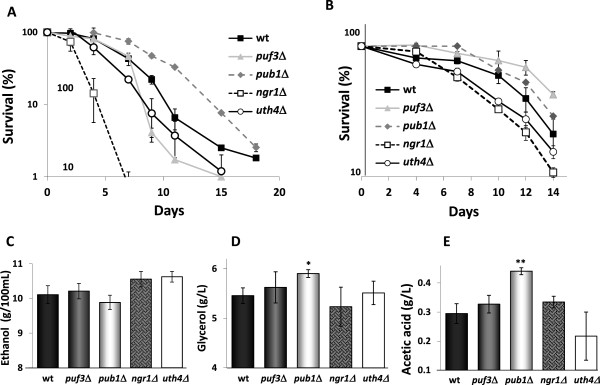
**Deletion of mRNA binding proteins alters lifespan under different growth conditions. **Genes were deleted from haploid wine yeast C9. **A**) The CLS experiments in laboratory medium SC. Viability after three days in SC is taken as 100% survival. Aliquots of each culture were taken, diluted, plated and counted over time. **B**) The CLS plots during the grape juice fermentation of the same deletion strains. Other conditions as in Figure 1C. Production of ethanol (**C**), acetic acid (**D**) and glycerol (**E**) in the vinifications described in Panel (**B**). Experiments were done in triplicate and the mean and standard deviation are provided. *p<0.05, **p<0.01, unpaired t-test, two-tailed.

Next we focused on a different family of proteins with an impact on longevity, RNA-binding proteins, which may control many different processes at the posttranscriptional level. Uth4/Puf5 was one of the first age-related genes to be described [15]. In a global analysis of CLS, Puf3 (an Uth4 homolog) and Pub1 were two RNA binding proteins whose deletion increased life span [41]. Ngr1 was another RNA binding protein seen to be required for stationary phase survival [42]. We deleted all four genes in haploid wine strain C9 [43]. First, we tested the effect of the deletions on CLS under standard laboratory conditions in SC medium (Figure 4A). *NGR1* mutation brought about a sharp drop in viability, indicating its positive role in CLS in wine yeast. Uth4, which is required for replicative life span under laboratory strains, was also required to achieve full CLS in wine yeasts. In accordance with what happened in laboratory strains, the *PUB1* deletion extended CLS. However, the *PUF3* deletion, that in some experiments extends CLS [41] and in other does not affect it [44], unexpectedly shortened it (Figure 4A). That suggest an influence of genetic background on Puf3 function. Puf3 controls the levels of the mRNAs coding for mitochondrial proteins, suggesting that mitochondria may act in *S*. *cerevisiae* laboratory and wine strains in different ways.

The same strains were used to conduct fermentations on natural red grape juice. All the mutants completed fermentation successfully (Additional file 2: Figure S2A). The death profile at the end of fermentation (as seen in Figure 1B) is shown in Figure 4B. Consistently with the results obtained in the laboratory medium, *NGR1* and *UTH4* deletions shortened CLS, while deletion *PUB1* slightly prolonged it. However, the effect of *PUF3* deletion changed depending on the medium as its deletion lengthened CLS in grape juice (Figure 4B), which is the opposite effect seen in SC medium, but the same effect described for laboratory strains [41]. Due to the role of Puf3 in mitochondrial function regulation, the low oxygen level during grape juice fermentation must impose different conditions to mitochondria than the growth in low glucose in laboratory media, where respiration is necessary for longevity. Metabolites were monitored at the end of fermentation (Figures 4C, D, E). Ethanol production did not change significantly (Figure 4C), but the *PUB1* deletion increased glycerol (Figure 4D) and acetic acid (Figure 4E). Pub1 binds the mRNA of glycerol 3-phosphate dehydrogenase *GPD2*[45], which could contribute to this increased production.

**Figure 5:**
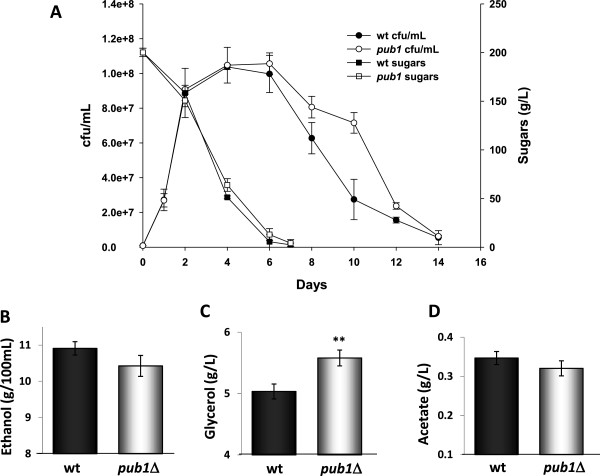
**The *PUB1 *deletion increases glycerol production and extends CLS during winemaking. A**) Natural grape juice fermentations of the *PUB1 *deletion strain and its parental strain L2056. Cell viability over time was measured as cfu/mL and fermentation evolution was followed by measuring sugar consumption. Ethanol (**B**), acetic acid (**C**) and glycerol (**D**) production of the fermentation described in Panel **A**. Experiments were done in triplicate and the mean and standard deviation are provided. **p<0.01, unpaired t-test, two-tailed.

As the *PUB1* deletion extended longevity in a variety of growth media and it increased the levels of glycerol, a metabolite with a positive impact on wine properties, we performed the deletion of the two copies of *PUB1* present in industrial wine strain EC1118, a widely used commercial strain with a relatively short CLS if compared to other strains [12]. The null *pub1*Δ mutant was able to complete natural grape juice fermentation (Figure 5A), although the sugar consumption rate was slightly slower at later fermentation stages. Cell growth was normal and life span extended once sugars had been exhausted in the deletion mutant, as it happened in the previous experiments. Metabolite production at the end of this fermentation was measured (Figures 5B,C,D). Ethanol and acetic acid production were mildly lower in the mutant strain, but in this case, the drop in acetic acid was not significant (Figure 5C), unlike what happened in the C9 strain. Therefore, genetic background influenced the output of this mutation. However, glycerol production was significantly higher in the strain carrying the *PUB1* deletion (Figure 5D), as was the case in the C9 background, indicating that Pub1 plays a relevant role in glycerol production, and that its manipulation is an efficient strategy to increase the concentration of this valuable metabolite.

**Figure 6:**
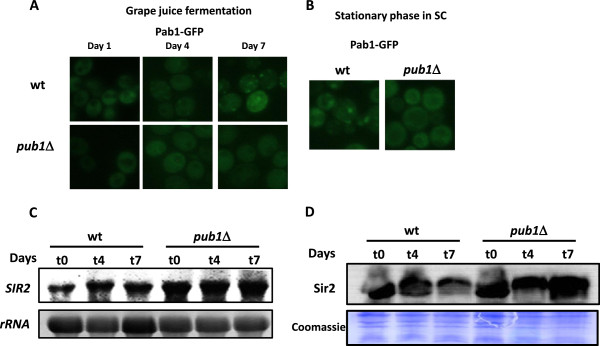
**Pub1 is necessary for stress granule (SG) formation and regulates Sir2 expression during grape juice fermentation. A**) SG formation during grape juice fermentation. A Pab1-GFP fusion was inserted in the EC1118 wild type strain and its *pub1*Δ derivative and its fluorescence was observed at days 1, 4 and 7 of grape juice fermentation. **B**) SG formation at stationary phase in SC medium. The same strains were grown for three days in SC, the reference point for CLS experiments in laboratory conditions. **C**) Northern blot analysis of the *SIR2 *mRNA levels during grape juice fermentation for the EC1118 strain and its *pub1*Δ derivative. rRNA is used as the loading control. **D**) Western blot of Sir2 protein in the same conditions of section C). Coomassie blue staining was used as the loading control.

Pub1 and Ngr1 take part in cytoplasmic aggregates known as stress granules (SG) during glucose starvation and other stress conditions [46]. SG contain mRNA and translation factors, such as the poly(A) binding protein Pab1. In fact, the *PUB1* deletion blocks the formation of such SG in glucose starvation [47], but not in the presence of translation inhibitor sodium azide [48]. However *NGR1* deletion has no effect in SG formation [47]. We tagged with GFP the SG specific protein Pab1 in wine yeast EC1118 and its *pub1*Δ derivative, and SG formation was followed during grape juice fermentation (Figure 6A). Pab1 has a uniform cytosolic distribution during the first days of fermentation, and SG do not appear in the wild type strain up to day four, when cells reached stationary phase and growth ceased (see Figure 5A). In this conditions, SG are not formed in the mutant strain (Figure 6A). At day 7, when fermentation is finished, SG are bigger in the wild type strain, but in the *pub1*Δ strain they are absent or very faint. Therefore, stress granules are formed in the late phases of winemaking, and their formation depends on Pub1. Next we observed SG formation in the laboratory medium SC where CLS experiments are performed. In stationary phase after three days in this medium (that correspond to day 0 in Figure 4A), stress granules are formed (Figure 6B), although they tend to be fewer (many times there is only one per cell) than the ones observed in grape juice fermentation. *PUB1* deletion cause also SG disruption in this condition. Therefore, stress granule formation is not necessary to achieve full life span, and they may be even deleterious for longevity, as *PUB1* deletion extends CLS and reduce SG in both conditions tested. Maybe SG are important to survival after short stresses but sequestration of translation factors may impair long-term survival.

Pub1 is also a gene expression regulator that has been linked to the control of mRNA stability and translation [45]. We have previously seen that *SIR2* mRNA levels change when cells are exposed to acetaldehyde, suggesting that they are controlled by the metabolic status of the cell [49]. RNA was extracted during grape juice fermentation on days 1, 4 and 7, and the level of *SIR2* mRNA was measured by Northern blot (Figure 6C). We saw that *SIR2* was up-regulated in the *pub1*Δ mutant throughout fermentation. In a global analysis, *SIR2* mRNA was not detected as a binding target of the Pub1 protein [45], so the effect of Pub1 on *SIR2* mRNA levels may be indirect. The deletion of other mRNA binding proteins had no impact on the *SIR2* mRNA levels (data not shown). We measured Sir2 protein levels during fermentation by western blot (Figure 6D) and found that protein levels are also higher in the *PUB1* deletion strain, in similar levels, suggesting that most of the effect of Pub1 on *SIR2* gene expression is acting at the RNA level, not at translation, as protein levels follow mRNA abundance. In any case that elevated Sir2 levels may contribute to extend longevity during grape juice fermentation in the *PUB1* deletion strain.

### Apoptosis plays an unexpected positive role during winemaking

**Figure 7:**
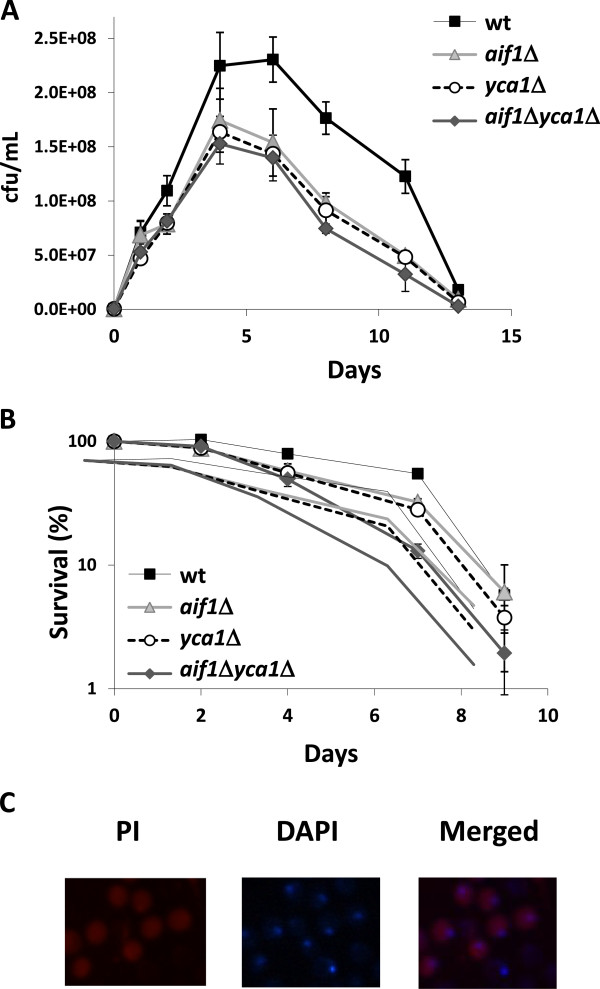
**Deletion of apoptosis-related genes shortens CLS under winemaking conditions. **The deletion mutants of *YCA1*, *AIF1*, or both, on the C9 strain were used for grape juice fermentations. **A**) Cell viability over time was measured as cfu/mL. **B**) Survival curves after taking day 4 of fermentation as 100% survival. Experiments were done in triplicate and the mean and standard deviation are provided. **C**) C9 strain after 8 days of fermentation was co-stained with propidium iodide (PI) and DAPI, and observed under a fluorescence microscopy. A merged image is also shown.

Finally, we tested the effect of the deletion of apoptosis-related genes during grape juice fermentation. We chose yeast caspase gene *YCA1* and mitochondria-related apoptosis-inducing factor *AIF1* to test different branches of the process. Both deletions extended CLS under laboratory conditions [50,51]. We performed independent and double deletions in haploid wine yeast strain C9 to construct the double mutant *yca1*Δ *aif1*Δ easily. These strains were used as starters during natural grape juice fermentation. Mutants were able to complete fermentation and showed no defect upon sugar consumption (Additional file 2: Figure S2B). The growth profiles of all strains are shown in Figure 7A. All the mutant strains presented a slower cell growth rate and reached lower maximal cell density. If the cell count on day 4 was taken as 100% viability, a death profile could be obtained (Figure 7B). Surprisingly, both single mutants presented a shortened life span under winemaking conditions, suggesting that both the caspase-dependent and caspase-independent (and mitochondrial-dependent) branches of apoptosis do not promote cell death under these conditions. The fact that the double mutation effect was additive reinforces the idea that both proteins not only act in different pathways, but also play a positive role in CLS under winemaking conditions. As previously described by our group for other pathways, such as autophagy, the effect of apoptotic machinery on winemaking is the opposite to the effect observed under laboratory conditions. Growth conditions during winemaking (high sugar, low nitrogen) differ from laboratory growth conditions (low sugar, high nitrogen), which may affect the trigger of programmed death mechanisms [52]. Low oxygen condition does not seem relevant as the blockage of both mitochondrial- dependent and -independent branches of apoptosis displays similar effects under winemaking conditions.

In previous work we have observed that at the end of grape juice fermentation cells are stained by the necrosis-specific dye propidium iodide (PI) [9,12]. At day 8 of natural grape juice fermentation carried out by the wild type wine strain C9, death cells are stained in red by PI (Figure 7C). DAPI staining of nuclei at the same time indicate that most nuclei are intact at the end of fermentation (Figure 7C). Nuclear fragmentation is a marker of apoptosis that can be detected by DAPI staining [53]. Merged images showed that cells with damaged membrane integrity (PI stained) have intact nuclei, suggesting that cells at the end of fermentation suffer a necrotic death. Mutants in apoptosis factors showed the same profile (data not shown), reinforcing the idea that death do not happen through apoptosis in winemaking conditions.

## Conclusions

In this work, we studied the effects of genetic manipulation of a variety of age-related genes under winemaking conditions. Chronological life span (CLS) during grape juice fermentation can be extended by overexpressing sirtuin gene *SIR2* or by deleting RNA-binding proteins genes *PUB1* and *PUF3*. Pub1 is necessary for stress granule formation in winemaking conditions and during standard CLS experiments in SC medium. Both sirtuin *HST3* overexpression and RNA-binding protein gene *NGR1* deletion increase death, therefore promoting cell lysis, which are useful processes to improve the organoleptic properties of wine or to promote malolactic bacteria growth. Aging is closely linked to metabolism, and many of those genetic manipulations lead to changes in the metabolites produced during wine fermentation. The ability of the *HST3* overexpressing strain to produce high ethanol and low acetic acid is an interesting property from a commercial perspective. There is a correlation between ethanol production and CLS as an increase in the *HST3* level leads to high ethanol and shortened longevity, and the strain displaying a high *SIR2* expression presents increased CLS, with slightly lower ethanol production. Conversely to observations under laboratory conditions [21], acetic acid is not the main cause of aging under winemaking conditions. Glycerol has been identified as a longevity inducer under laboratory conditions [23], and may explain the longer longevity of the *pub1*Δ strain. It is worth noting that all the manipulated strains were able to complete fermentation and that they all consumed sugars completely, and usually at a similar rate to the parental strain. Therefore, this aspect of metabolism is very robust and cells seem to adapt to changes in metabolism-related genes in order to keep the fermentation pathway at an optimal speed, although the proportion of some metabolites could change in these manipulated strains.

## Methods

### Yeast strains and growth media

Additional file 3: Table S1 lists the industrial wine yeasts used in this work. Haploid strain C9 was a gift from M. Walker [43]. Additional file 4: Table S2 lists the oligonucleotides employed to amplify deletion and over-expression cassettes and to check transformants. Gene over-expression strains were obtained by following a promoter replacement strategy developed in our laboratory [32]. Plasmid pkanMX-SPI1p [32] was used to PCR-amplify the cassettes for gene over-expressions under the control of the *SPI1* gene promoter. In order to express genes under the control of the *MET17* promoter, plasmid pkanMX-MET17p was constructed by amplifying the MET17 promoter by PCR using oligonucleotides MET17a and MET17b (see Additional file 4: Table S2) and by cloning the amplified fragment at the *Eco*RV site of plasmid pUG6 [54]. Gene disruptions were performed by using recyclable selection marker *loxPkanMXloxP* from plasmid pUG6 following the protocol of Güldener et al. [54]. The marker was eliminated by transforming with the cre recombinase-containing plasmid YEp351-cre-cyh according to Delneri et al. [55]. For GFP fusions, plasmid pFA6a-GFP(S65T)-*kanMX6*[56] was used as a template to C-terminal tagging of *PAB1* gene. Yeast transformations were carried out by the lithium acetate method [57].

For yeast growth under laboratory conditions, YPD medium (1% yeast extract, 2% bactopeptone, 2% glucose) and SC medium (0.17% yeast nitrogen base, 0.5% ammonium sulfate, 2% glucose and 0.2% drop-out mix with all the amino acids) were used [58]. Selective plates contained 20 μg mL^-1^geneticine or 0.1 μg mL^-1^cycloheximide. Red grape juice (Bobal variety) was a gift from Bodegas Murviedro and was sterilized overnight with 500 μg/L of dimethyldicarbonate. Synthetic grape juice MS300 was made as previously described [9,59].

### Grape juice fermentations, chronological life span measurements and stress conditions

For the microvinification experiments, cells from the 2-day cultures in YPD were inoculated at a final concentration of 10^6^ cells/mL in filled-in conical centrifuge tubes with 50 mL of grape juice. Incubation was done at very low shaking at 22°C. Evolution of vinifications was followed by determining cell viability by diluting, plating and counting colony forming units (cfu) and sugar consumption, as previously described [60]. Survival plots were drawn by taking the highest cell viability point (around 2-4 days) as 100% survival. Dry biomass measurement was carried out using the filtration method [61] and weighting the filters after drying at 65°C overnight.

Under laboratory conditions, the CLS experiments were performed as follows: precultures of selected strains were grown overnight on YPD and inoculated in SC media at an OD_600_ of 0.1. After day 3 of growth at 30°C, aliquots were taken, diluted and plated. Colonies were counted and percentage of survival was calculated taken day 3 of growth as 100% survival. Ethanol tolerance was measured in stationary cultures on YPD by adding 10% ethanol for 1 h. Cell viability was measuring by diluting, plating and counting cfu.

### Metabolite determinations

Consumption of sugars during fermentation was measured by their reaction to DNS (dinitro-3,5-salycilic acid) following a modified version of Miller’s method [62]. Ethanol, acetic acid and glycerol were measured with the kits provided by r-Biopharm following the manufacturer’s instructions.

### Western and Northern blots

For the Western blot analysis, cells were broken with a volume of glass beads in a buffer containing Tris-HCl 0.1M pH 7.5, NaCl 0.5M, MgCl_2_ 0.1M, NP40 1% (v/v), PMSF 10 mM and protease inhibitors (complete Mini, EDTA-free from Roche). Protein concentration was measured by the Bradford method [63] using the Bio-Rad Protein assay following the manufacturer’s instructions. Extracts were diluted in loading buffer (Tris-HCl 240 mM pH 6.8, SDS 8%, glycerol 40%, β-mercaptoethanol 10%).

To conduct the Western blot analysis, SDS-PAGE total protein separations were done in an Invitrogen mini-gel device and were blotted onto PVDF membranes. The anti-Sir2 antibodies were obtained from Santa Cruz Biotechnology (Santa Cruz, USA). The ECL Western blotting detection system (Amersham) was used following the manufacturer’s instructions.

Total RNA isolation and formaldehyde denaturing agarose gels and blotting were carried out as previously described [64]. The *SIR2* probe was obtained by PCR and digoxigenine labeling using the PCR DIG labelling mix from Roche according to the manufacturer’s instructions. Probe detection was achieved by chemiluminescence using the DIG Northern Stater and CDP-Star kits by Roche, and was detected in an LAS-1000 device by Fujifilm.

### Microscopy methods

500 μl of cells were washed in PBS buffer and 5 μl of a 1 mg/mL stock solution of propidium iodide and 1 μl of a 1 mg/mL stock solution of DAPI (in dimethylformamide) were added to be then incubated in darkness for 20 min. Cells were washed in PBS and visualized. GFP-labelled cells were observed directly. Cells were visualized with the right filter under a Nikon eclipse 90i fluorescence microscope.

## Competing interests

The authors declare that they have no competing interests.

## Authors’ contributions

HO carried out the experimental methods and analyzed the data. EM contributed to the experimental design. AA designed the experiments and wrote the manuscript. All the authors discussed the data, and read, reviewed and approved the final manuscript.

## Supplementary Material

Additional file 1: Figure S1 Fermentation in synthetic grape juice of *HST3 *overproducing strains (A) Dry biomass production. (B) Ethanol production. *p<0.05, ***p<0.005, unpaired t-test, two-tailed. Click here for file

Additional file 2: Figure S2 Sugar consumption during grape juice fermentation by mutants in mRNA binding proteins (A) and apoptosis –related genes (B). The data reflect the experiments shown in Figure 4B and Figure 7A respectively. Click here for file

Additional file 3: Table S1 Yeast strains used in this work. Click here for file

Additional file 4: Table S2 Oligonucleotides used in this work. Click here for file

## References

[B1] Carnes BA What is lifespan regulation and why does it exist? Biogerontology 2011 12 367 374 10.1007/s10522-011-9338-3 21512719

[B2] Kaeberlein M Lessons on longevity from budding yeast Nature 2010 464 513 519 10.1038/nature08981 20336133 PMC3696189

[B3] Powell CD Van Zandycke SM Quain DE Smart KA Replicative ageing and senescence in Saccharomyces cerevisiae and the impact on brewing fermentations Microbiology 2000 146 Pt 5 1023 1034 10832629 10.1099/00221287-146-5-1023

[B4] Basso LC de Amorim HV de Oliveira AJ Lopes ML Yeast selection for fuel ethanol production in Brazil FEMS Yeast Res 2008 8 1155 1163 10.1111/j.1567-1364.2008.00428.x 18752628

[B5] Ribéreau-Gayon P Dubourdieu D Donèche B Handbook of enology 2006 2 Chichester, West Sussex, England; Hoboken, NJ: John Wiley

[B6] Qin H Lu M Natural variation in replicative and chronological life spans of Saccharomyces cerevisiae Exp Gerontol 2006 41 448 456 10.1016/j.exger.2006.01.007 16516427

[B7] Fornairon-Bonnefond C Salmon JM Impact of oxygen consumption by yeast lees on the autolysis phenomenon during simulation of wine aging on lees J Agric Food Chem 2003 51 2584 2590 10.1021/jf0259819 12696941

[B8] Alexandre H Costello PJ Remize F Guzzo J Guilloux-Benatier M Saccharomyces cerevisiae-Oenococcus oeni interactions in wine: current knowledge and perspectives Int J Food Microbiol 2004 93 141 154 10.1016/j.ijfoodmicro.2003.10.013 15135953

[B9] Orozco H Matallana E Aranda A Two-carbon metabolites, polyphenols and vitamins influence yeast chronological life span in winemaking conditions Microb Cell Fact 2012 11 104 10.1186/1475-2859-11-104 22873488 PMC3503821

[B10] Harman D Aging: a theory based on free radical and radiation chemistry J Gerontol 1956 11 298 300 10.1093/geronj/11.3.298 13332224

[B11] Fabrizio P Longo VD The chronological life span of Saccharomyces cerevisiae Methods Mol Biol 2007 371 89 95 10.1007/978-1-59745-361-5_8 17634576

[B12] Orozco H Matallana E Aranda A Oxidative stress tolerance, adenylate cyclase, and autophagy are key players in the chronological life span of saccharomyces cerevisiae during winemaking Appl Environ Microbiol 2012 78 2748 2757 10.1128/AEM.07261-11 22327582 PMC3318821

[B13] Blagosklonny MV Aging: ROS or TOR Cell Cycle 2008 7 3344 3354 10.4161/cc.7.21.6965 18971624

[B14] Kennedy BK Austriaco NR Jr Zhang J Guarente L Mutation in the silencing gene SIR4 can delay aging in S. cerevisiae Cell 1995 80 485 496 10.1016/0092-8674(95)90499-9 7859289

[B15] Kennedy BK Gotta M Sinclair DA Mills K McNabb DS Murthy M Pak SM Laroche T Gasser SM Guarente L Redistribution of silencing proteins from telomeres to the nucleolus is associated with extension of life span in S. cerevisiae Cell 1997 89 381 391 10.1016/S0092-8674(00)80219-6 9150138

[B16] Longo VD Kennedy BK Sirtuins in aging and age-related disease Cell 2006 126 257 268 10.1016/j.cell.2006.07.002 16873059

[B17] Dang W Steffen KK Perry R Dorsey JA Johnson FB Shilatifard A Kaeberlein M Kennedy BK Berger SL Histone H4 lysine 16 acetylation regulates cellular lifespan Nature 2009 459 802 807 10.1038/nature08085 19516333 PMC2702157

[B18] Fabrizio P Gattazzo C Battistella L Wei M Cheng C McGrew K Longo VD Sir2 blocks extreme life-span extension Cell 2005 123 655 667 10.1016/j.cell.2005.08.042 16286010

[B19] Lin YY Lu JY Zhang J Walter W Dang W Wan J Tao SC Qian J Zhao Y Boeke JD Protein acetylation microarray reveals that NuA4 controls key metabolic target regulating gluconeogenesis Cell 2009 136 1073 1084 10.1016/j.cell.2009.01.033 19303850 PMC2696288

[B20] Ralser M Michel S Breitenbach M Sirtuins as regulators of the yeast metabolic network Front Pharmacol 2012 3 32 22408620 10.3389/fphar.2012.00032 PMC3296958

[B21] Burtner CR Murakami CJ Kennedy BK Kaeberlein M A molecular mechanism of chronological aging in yeast Cell Cycle 2009 8 1256 1270 10.4161/cc.8.8.8287 19305133 PMC2746416

[B22] Murakami CJ Wall V Basisty N Kaeberlein M Composition and acidification of the culture medium influences chronological aging similarly in vineyard and laboratory yeast PLoS One 2011 6 e24530 10.1371/journal.pone.0024530 21949725 PMC3176285

[B23] Wei M Fabrizio P Madia F Hu J Ge H Li LM Longo VD Tor1/Sch9-regulated carbon source substitution is as effective as calorie restriction in life span extension PLoS Genet 2009 5 e1000467 10.1371/journal.pgen.1000467 19424415 PMC2669710

[B24] Fontana L Partridge L Longo VD Extending healthy life span–from yeast to humans Science 2010 328 321 326 10.1126/science.1172539 20395504 PMC3607354

[B25] Lin SJ Defossez PA Guarente L Requirement of NAD and SIR2 for life-span extension by calorie restriction in Saccharomyces cerevisiae Science 2000 289 2126 2128 10.1126/science.289.5487.2126 11000115

[B26] Couzin-Frankel J Genetics. Aging genes: the sirtuin story unravels Science 2011 334 1194 1198 10.1126/science.334.6060.1194 22144592

[B27] Burnett C Valentini S Cabreiro F Goss M Somogyvari M Piper MD Hoddinott M Sutphin GL Leko V McElwee JJ Absence of effects of Sir2 overexpression on lifespan in C. elegans and Drosophila Nature 2011 477 482 485 10.1038/nature10296 21938067 PMC3188402

[B28] Orozco H Matallana E Aranda A Wine yeast sirtuins and Gcn5p control aging and metabolism in a natural growth medium Mech Ageing Dev 2012 133 348 358 10.1016/j.mad.2012.03.013 22738658

[B29] Herker E Jungwirth H Lehmann KA Maldener C Frohlich KU Wissing S Buttner S Fehr M Sigrist S Madeo F Chronological aging leads to apoptosis in yeast J Cell Biol 2004 164 501 507 10.1083/jcb.200310014 14970189 PMC2171996

[B30] Fabrizio P Battistella L Vardavas R Gattazzo C Liou LL Diaspro A Dossen JW Gralla EB Longo VD Superoxide is a mediator of an altruistic aging program in Saccharomyces cerevisiae J Cell Biol 2004 166 1055 1067 10.1083/jcb.200404002 15452146 PMC2172019

[B31] Ruckenstuhl C Carmona-Gutierrez D Madeo F The sweet taste of death: glucose triggers apoptosis during yeast chronological aging Aging (Albany NY) 2010 2 643 649 21076182 10.18632/aging.100223 PMC2993794

[B32] Cardona F Carrasco P Perez-Ortin JE del Olmo M Aranda A A novel approach for the improvement of stress resistance in wine yeasts Int J Food Microbiol 2007 114 83 91 10.1016/j.ijfoodmicro.2006.10.043 17187885

[B33] Rossignol T Dulau L Julien A Blondin B Genome-wide monitoring of wine yeast gene expression during alcoholic fermentation Yeast 2003 20 1369 1385 10.1002/yea.1046 14663829

[B34] Puig S Perez-Ortin JE Stress response and expression patterns in wine fermentations of yeast genes induced at the diauxic shift Yeast 2000 16 139 148 10.1002/(SICI)1097-0061(20000130)16:2<139::AID-YEA512>3.0.CO;2-J 10641036

[B35] Kaeberlein M McVey M Guarente L The SIR2/3/4 complex and SIR2 alone promote longevity in Saccharomyces cerevisiae by two different mechanisms Genes Dev 1999 13 2570 2580 10.1101/gad.13.19.2570 10521401 PMC317077

[B36] Koutelou E Hirsch CL Dent SY Multiple faces of the SAGA complex Curr Opin Cell Biol 2010 22 374 382 10.1016/j.ceb.2010.03.005 20363118 PMC2900470

[B37] Starai VJ Takahashi H Boeke JD Escalante-Semerena JC Short-chain fatty acid activation by acyl-coenzyme A synthetases requires SIR2 protein function in Salmonella enterica and Saccharomyces cerevisiae Genetics 2003 163 545 555 12618394 10.1093/genetics/163.2.545 PMC1462443

[B38] Martorell P Forment JV de Llanos R Monton F Llopis S Gonzalez N Genoves S Cienfuegos E Monzo H Ramon D Use of Saccharomyces cerevisiae and Caenorhabditis elegans as model organisms to study the effect of cocoa polyphenols in the resistance to oxidative stress J Agric Food Chem 2011 59 2077 2085 10.1021/jf104217g 21288028

[B39] Fabrizio P Liou LL Moy VN Diaspro A Valentine JS Gralla EB Longo VD SOD2 functions downstream of Sch9 to extend longevity in yeast Genetics 2003 163 35 46 12586694 10.1093/genetics/163.1.35 PMC1462415

[B40] Orozco H Matallana E Aranda A Oxidative stress tolerance, adenylate cyclase and autophagy are key players in yeast chronological life span during winemaking Appl Environ Microbiol 2012 78 2748 2757 10.1128/AEM.07261-11 22327582 PMC3318821

[B41] Powers RW III Kaeberlein M Caldwell SD Kennedy BK Fields S Extension of chronological life span in yeast by decreased TOR pathway signaling Genes Dev 2006 20 174 184 10.1101/gad.1381406 16418483 PMC1356109

[B42] Martinez MJ Roy S Archuletta AB Wentzell PD Anna-Arriola SS Rodriguez AL Aragon AD Quinones GA Allen C Werner-Washburne M Genomic analysis of stationary-phase and exit in Saccharomyces cerevisiae: gene expression and identification of novel essential genes Mol Biol Cell 2004 15 5295 5305 10.1091/mbc.E03-11-0856 15456898 PMC532011

[B43] Walker ME Gardner JM Vystavelova A McBryde C de Barros Lopes M Jiranek V Application of the reuseable, KanMX selectable marker to industrial yeast: construction and evaluation of heterothallic wine strains of Saccharomyces cerevisiae, possessing minimal foreign DNA sequences FEMS Yeast Res 2003 4 339 347 10.1016/S1567-1356(03)00161-2 14654439

[B44] Chatenay-Lapointe M Shadel GS Repression of mitochondrial translation, respiration and a metabolic cycle-regulated gene, SLF1, by the yeast Pumilio-family protein Puf3p PLoS One 2011 6 e20441 10.1371/journal.pone.0020441 21655263 PMC3105058

[B45] Duttagupta R Tian B Wilusz CJ Khounh DT Soteropoulos P Ouyang M Dougherty JP Peltz SW Global analysis of Pub1p targets reveals a coordinate control of gene expression through modulation of binding and stability Mol Cell Biol 2005 25 5499 5513 10.1128/MCB.25.13.5499-5513.2005 15964806 PMC1156976

[B46] Balagopal V Parker R Polysomes, P bodies and stress granules: states and fates of eukaryotic mRNAs Curr Opin Cell Biol 2009 21 403 408 10.1016/j.ceb.2009.03.005 19394210 PMC2740377

[B47] Buchan JR Muhlrad D Parker R P bodies promote stress granule assembly in Saccharomyces cerevisiae J Cell Biol 2008 183 441 455 10.1083/jcb.200807043 18981231 PMC2575786

[B48] Buchan JR Yoon JH Parker R Stress-specific composition, assembly and kinetics of stress granules in Saccharomyces cerevisiae J Cell Sci 2011 124 228 239 10.1242/jcs.078444 21172806 PMC3010191

[B49] Aranda A del Olmo ML Exposure of Saccharomyces cerevisiae to acetaldehyde induces sulfur amino acid metabolism and polyamine transporter genes, which depend on Met4p and Haa1p transcription factors, respectively Appl Environ Microbiol 2004 70 1913 1922 10.1128/AEM.70.4.1913-1922.2004 15066780 PMC383134

[B50] Madeo F Herker E Maldener C Wissing S Lachelt S Herlan M Fehr M Lauber K Sigrist SJ Wesselborg S Frohlich KU A caspase-related protease regulates apoptosis in yeast Mol Cell 2002 9 911 917 10.1016/S1097-2765(02)00501-4 11983181

[B51] Wissing S Ludovico P Herker E Buttner S Engelhardt SM Decker T Link A Proksch A Rodrigues F Corte-Real M An AIF orthologue regulates apoptosis in yeast J Cell Biol 2004 166 969 974 10.1083/jcb.200404138 15381687 PMC2172025

[B52] Ring J Sommer C Carmona-Gutierrez D Ruckenstuhl C Eisenberg T Madeo F The metabolism beyond programmed cell death in yeast Exp Cell Res 2012 318 1193 1200 10.1016/j.yexcr.2012.03.019 22480867 PMC3396845

[B53] Laun P Pichova A Madeo F Fuchs J Ellinger A Kohlwein S Dawes I Frohlich KU Breitenbach M Aged mother cells of Saccharomyces cerevisiae show markers of oxidative stress and apoptosis Mol Microbiol 2001 39 1166 1173 10.1111/j.1365-2958.2001.02317.x 11251834

[B54] Guldener U Heck S Fielder T Beinhauer J Hegemann JH A new efficient gene disruption cassette for repeated use in budding yeast Nucleic Acids Res 1996 24 2519 2524 10.1093/nar/24.13.2519 8692690 PMC145975

[B55] Delneri D Tomlin GC Wixon JL Hutter A Sefton M Louis EJ Oliver SG Exploring redundancy in the yeast genome: an improved strategy for use of the cre-loxP system Gene 2000 252 127 135 10.1016/S0378-1119(00)00217-1 10903444

[B56] Longtine MS McKenzie A III Demarini DJ Shah NG Wach A Brachat A Philippsen P Pringle JR Additional modules for versatile and economical PCR-based gene deletion and modification in Saccharomyces cerevisiae Yeast 1998 14 953 961 10.1002/(SICI)1097-0061(199807)14:10<953::AID-YEA293>3.0.CO;2-U 9717241

[B57] Gietz RD Woods RA Transformation of yeast by lithium acetate/single-stranded carrier DNA/polyethylene glycol method Methods Enzymol 2002 350 87 96 12073338 10.1016/s0076-6879(02)50957-5

[B58] Adams A Kaiser C Cold Spring Harbor Laboratory Methods in yeast genetics: a Cold Spring Harbor Laboratory course manual 1998 1997 Plainview, N.Y: Cold Spring Harbor Laboratory Press

[B59] Riou C Nicaud JM Barre P Gaillardin C Stationary-phase gene expression in Saccharomyces cerevisiae during wine fermentation Yeast 1997 13 903 915 10.1002/(SICI)1097-0061(199708)13:10<903::AID-YEA145>3.0.CO;2-1 9271106

[B60] Zuzuarregui A del Olmo ML Expression of stress response genes in wine strains with different fermentative behavior FEMS Yeast Res 2004 4 699 710 10.1016/j.femsyr.2004.01.008 15093773

[B61] Pringle JR Mor JR Methods for monitoring the growth of yeast cultures and for dealing with the clumping problem Methods Cell Biol 1975 11 131 168 1102845 10.1016/s0091-679x(08)60320-9

[B62] Robyt JF Whelan WJ Reducing value methods for maltodextrins. I. Chain-length dependence of alkaline 3,5-dinitrosalicylate and chain-length independence of alkaline copper Anal Biochem 1972 45 510 516 10.1016/0003-2697(72)90213-8 5060605

[B63] Bradford MM A rapid and sensitive method for the quantitation of microgram quantities of protein utilizing the principle of protein-dye binding Anal Biochem 1976 72 248 254 10.1016/0003-2697(76)90527-3 942051

[B64] Aranda A Perez-Ortin JE Moore C del Olmo ML Transcription termination downstream of the Saccharomyces cerevisiae FBP1 [changed from FPB1] poly(A) site does not depend on efficient 3'end processing RNA 1998 4 303 318 9510332 PMC1369619

