# Supplementary figures and images for: Genetic manipulation of longevity-related genes as a tool to regulate yeast life span and metabolite production during winemaking
Source: Microb Cell Fact. 2013 Jan 2;12:1. doi: 10.1186/1475-2859-12-1 (PMC3583744; doi:10.1186/1475-2859-12-1)

Figure S1

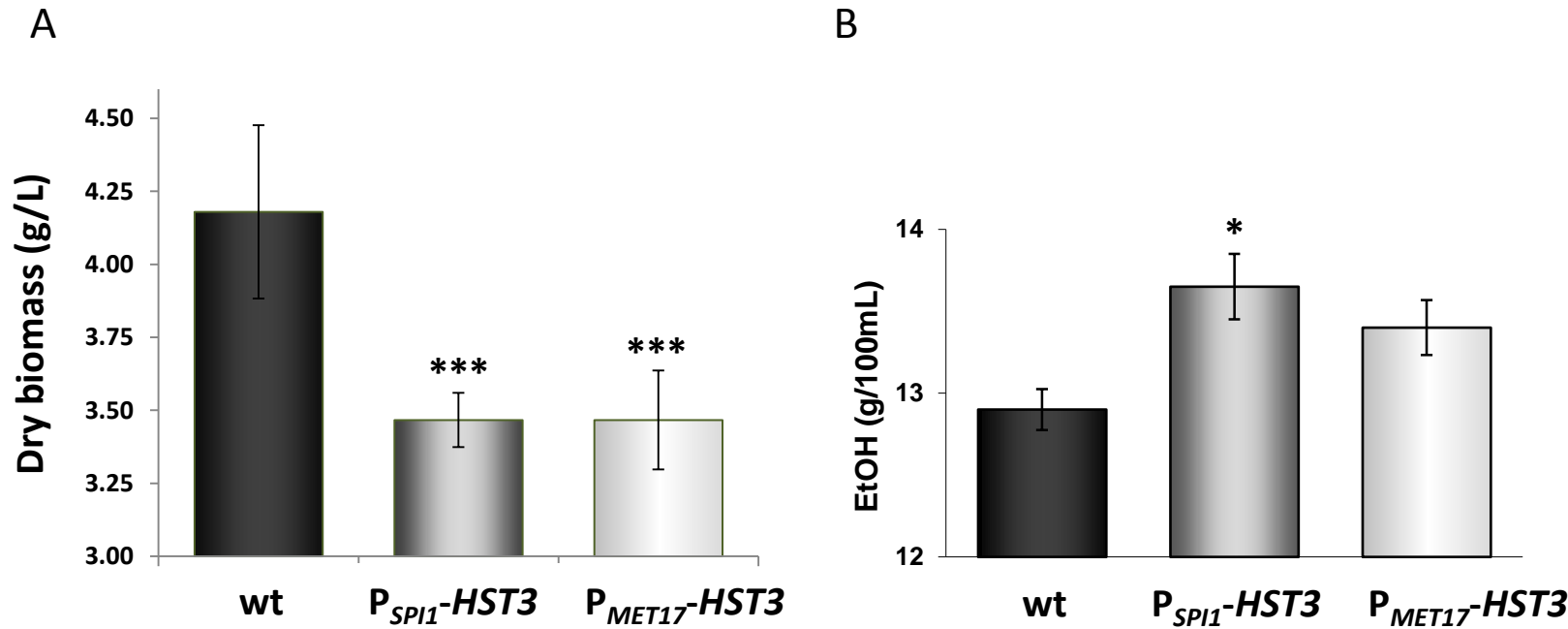

Supplement: Additional file 1: Figure S1 — Fermentation in synthetic grape juice of HST3 overproducing strains (A) Dry biomass production. (B) Ethanol production. *p<0.05, ***p<0.005, unpaired t-test, two-tailed. [file 1475-2859-12-1-S1.pdf]

Figure S2

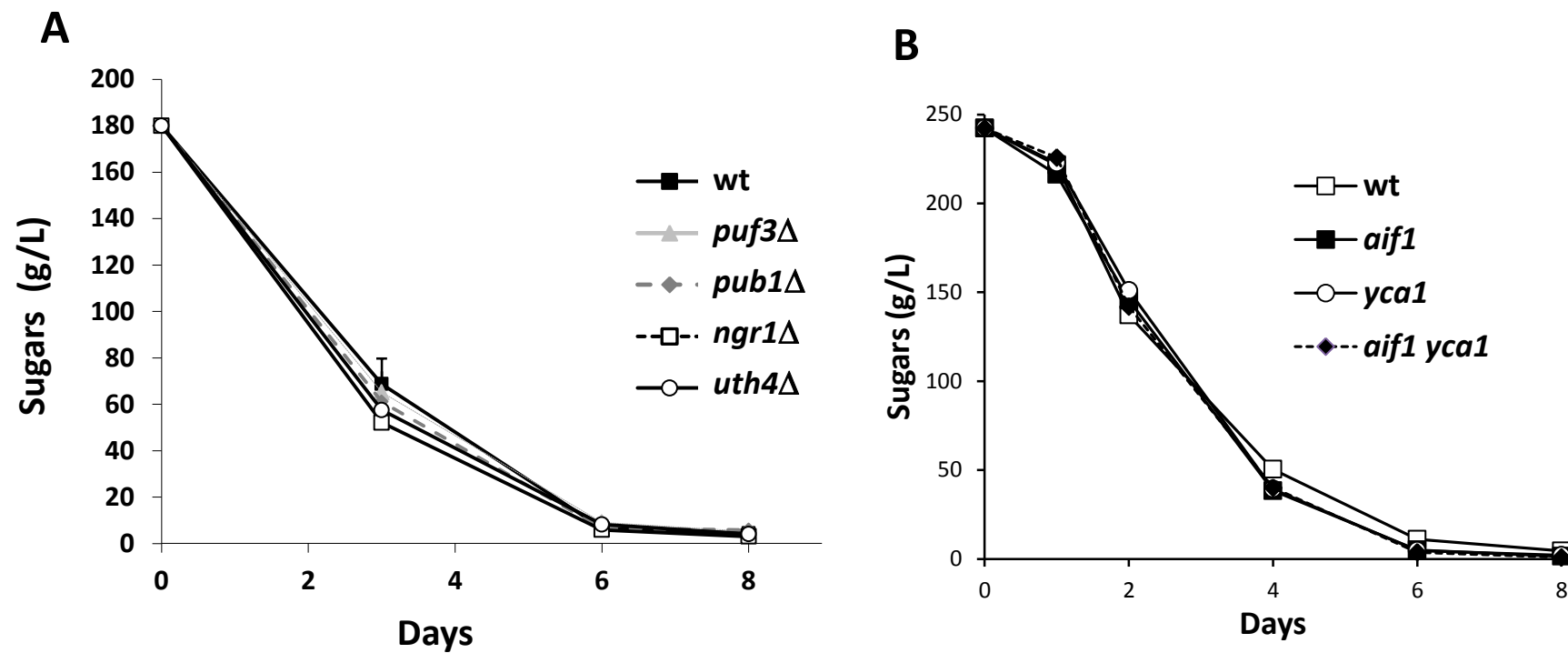

Supplement: Additional file 2: Figure S2 — Sugar consumption during grape juice fermentation by mutants in mRNA binding proteins (A) and apoptosis –related genes (B). The data reflect the experiments shown in Figure 4B and Figure 7A respectively. [file 1475-2859-12-1-S2.pdf]
