# Supplementary material for: Genetic manipulation of longevity-related genes as a tool to regulate yeast life span and metabolite production during winemaking
Source: Microb Cell Fact. 2013 Jan 2;12:1. doi: 10.1186/1475-2859-12-1 (PMC3583744; doi:10.1186/1475-2859-12-1)
Supplement: Additional file 4: Table S2 — Oligonucleotides used in this work. [file 1475-2859-12-1-S4.pdf]

Table S2. Oligonucleotides used in this work.

Oligo	Sequence	Use
SIR2g	ACCATCCCACATATGAAATACGCCGTATCA AAGACTAGCGTTCGTACGCTGCAGGTCGAC	Overexpression
SIR2e’	CGCTAGTCTTTGATACGGCGTATTTCATATGTGGGATGGTCATTATTAGTAATAGTACTG	*SPI1* overexpression
SIR2f	CGCTAGTCTTTGATACGGCGTATTTCATATGT GGGATGGTCATTGTATGGATGGGGGTAATAG	*MET17* overexpression
HST3g	TCTGTGGCGGGTATCCCTCAATTGCGGGTAACAATAAGTATcrTCGTACGCTGCAGGTCGAC	Overexpression
HST3f	CCGATCGACTGGCAGGTGGCGAGGGCGATACTGAAGTCATTATTAGTAATAGTACTG	*SPI1* overexpression
HST3h	CCGATCGACTGGCAGGTGGCGAGGGCGATACTGAAGTCATTGTATGGATGGGGGTAATAG	*MET17* overexpression
GCN5g	TATTGAAATTCTTACGCAAGATTTTTTATA GTTGGATATTTTCGTACGCTGCAGGTCGAC	Overexpression
GCN5f	CATCCAAGTGATCCTCTTCAATCTGATGTTTTGTGACCATTGTATGGATGGGGGTAATAG	*MET17* overexpression
SIR2d	AGTTGGTTTGAGCTCCATCG	Check
HST3c	ACTCAACATCACGAACGACG	Check
GCN5d	TTATTGGTCTCAGCCTGCTC	Check
SPI1e	CTCGAAGTTCCCAGATGCCC	Check
MET17c	CCT TGT CCA ATT GAA CAC GC	Check
MET17a	GGGATATCGAATCCCTTAGCTCTC	*MET17* promoter cloning
MET17b	GGGATATCATTGTATGGATGGGGG	*MET17* promoter cloning
Puf3a	CGCATTTAAATTTCTTCTGAATAACGCAATATTGCGGGTACGTACGCTGCAGGTCGAC	Deletion
Puf3b	GTAAAAAGTGAAAGGAGAACGATGATAACACTAATCACACCCACTAGTGGATCTGATATC	Deletion
Puf3c	TTTTCTGGTCATCTGGTCTC	Check
Pub1a	TTCATAATAAGAAGATTACCACATCTACTCTTTGTTCGCACGTACGCTGCAGGTCGAC	Deletion
Pub1b	GATTTGTAGGTTGCCTCTCTTTATTCTTTCTTTTTGTTCCCCACTAGTGGATCTGATATC	Deletion
Pub1c	TTGGCCGTCTTTCCTTGTCC	Check
Uth4a	GTACAGTAAGAAGGAAAGAAAAAGAAAGAAAAAAAAGTAACTAGTGGATCTGATATC	Deletion
Uth4b	AAATTTATAAATCAATTACGATTTTTCCAGTTTTCTCTTATGCGTACGCTGCAGGTCGAC	Deletion
Uth4c	AAGAACAGATCTGCTAGCCCCG	Check
Ngr1a	TCATATCCTTCGCCATCGATTTTTGCCTGAAAATTTACACACGTACGCTGCAGGTCGAC	Deletion
Ngr1b	ACTGCGGACAAGATTAAAATTTTCTTTTTTTGTCTTTGTCCACTAGTGGATCTGATATC	Deletion
Ngr1c	GTTCCAAAAAGCGTTCCCC	Check
Aif1a	AGGAAAGAGCAGAGAAAGGAAGAAAGAAATTGCAAAATATTTCGTACGCTGCAGGTCGAC	Deletion

Aif1b	TATATATATATACGCTGCAGTTCATATTTTAGTCTATTTTATAGGCCACTAGTGGATCTG	Deletion
Aif1c	CTGGCTGGACAAATGAAGCC	Check
Yca1a	TCAAAACTACCACCAAAGAAGACCGACTAGATTTACAATCTTCGTACGCTGCAGGTCGAC	Deletion
Yca1b	CAGTCTGAATACATCTACCAACGTACACATTCATATATTTATAGGCCACTAGTGGATCTG	Deletion
Yca1c	GGCTTCGCATTAATAGGAGC	Check
K2	GGGACAATTCAACGCGTCTG	Check
Sir2a	TTCTTTGTCCTCTTTGGCCG	Probe
Sir2b	AGTTGGTTTGAGCTCCATCG	Probe
MET17a	GGGATATCGAATCCCTTAGCTCTC	*MET17* promoter cloning
MET17b	GGGATATCATTGTATGGATGGGGG	*MET17* promoter cloning
PAB1-F2	GTCTTTCAAAAAGGAGCAAGAACAACAAACTGAGCAAGCTCGGATCCCCGGGTTAATTAA	GFP tagging
PAB1-R1	TAAGTTTGTTGAGTAGGGAAGTAGGTGATTACATAGAGCAGAATTCGAGCTCGTTTAAAC	GFP tagging
PAB1d	TGCGGTGATGAATAGCAAGG	*Check*
