# Supplementary material for: Genetic manipulation of longevity-related genes as a tool to regulate yeast life span and metabolite production during winemaking
Source: Microb Cell Fact. 2013 Jan 2;12:1. doi: 10.1186/1475-2859-12-1 (PMC3583744; doi:10.1186/1475-2859-12-1)
Supplement: Additional file 3: Table S1 — Yeast strains used in this work. [file 1475-2859-12-1-S3.pdf]

Table S1. Yeast strains used in this work

Strain	Genotype	Origin
L2056	Winestrain	Lallemand
L2056 P_{SPI1}-*SIR2*	L2056 P_{SPI1}-*SIR2-kanMX*	This work
L2056 P_{MET17}-*SIR2*	L2056 P_{MET17}-*SIR2-kanMX*	" "
L2056 P_{SPI1}-*HST3*	L2056 P_{SPI1}-*HST3-kanMX*	" "
L2056 P_{MET17}-*HST3*	L2056 P_{MET17}-*HST3-kanMX*	" "
L2056 P_{MET17}-*GCN5*	L2056 P_{MET17}-*GCN5-kanMX*	" "
C9	Mat a *ho*::*loxP*	JM Walker
C9 *puf3*	C9 *puf3::kanMX*	This work
C9 *pub1*	C9 *pub1::kanMX*	"
C9 *uth4*	C9 *uth4::kanMX*	"
C9 *aif1*	C9 *aif1::kanMX*	"
C9 *yca1*	C9 *yca1::kanMX*	"
C9 *aif1 yca1*	C9 *yca1::kanMX aif1*::*loxP*	"
EC1118	Wine strain	Lallemand
EC1118 *pub1*	EC1118 *pub1*::*loxP pub1*::*kanMX*	This work
EC1118 PAB1-GFP	EC1118 PAB1-GFP::*kanMX*	This work
EC1118 pub1 PAB1-GFP	EC1118 pub1::loxP pub1::kanMX PAB1-GFP::*kanMX*	This work
